# The progress of targeted therapy in advanced gastric cancer

**DOI:** 10.1186/2050-7771-1-32

**Published:** 2013-12-11

**Authors:** Miao-zhen Qiu, Rui-hua Xu

**Affiliations:** 1 Department of Medical Oncology, Sun Yat-Sen University Cancer Center, State Key Laboratory of Oncology in South China, Collaborative Innovation Center for Cancer Medicine, 651 Dong Feng Road East, Guangzhou 510060, China

## Abstract

Although palliative chemotherapy has been shown to prolong survival and improve quality of life, the survival of advanced gastric cancer (AGC) patients remains poor. With the advent of targeted therapy, many molecular targeted agents have been evaluated in clinical studies. Trastuzumab, an anti-HER2 monoclonal antibody, has shown activity against HER2-positive AGC and becomes the first targeted agent approved in AGC. Drugs that target epidermal growth factor receptor, including monoclonal antibody and tyrosine kinase inhibitor, do not bring survival benefit to patients with AGC. Additionally, vascular endothelial growth factor inhibitors are also under investigation. Ramucirumab has shown promising result. Other targeted agents are in preclinical or early clinical development, such as mammalian target of rapamycinm inhibitors and c-MET inhibitors.

## Introduction

Approximately one million people are diagnosed each year with gastric cancer, making it the fourth most common cancer and the second leading cause of cancer related death worldwide with an estimated 800,000 deaths caused by the disease [1]. The incidence of gastric cancer varies widely according to geographic region and is particularly common in eastern Asia [2]. According to the 2012 Chinese cancer registry annual report, gastric cancer is the third most common cancer and the second leading cause of cancer related death in China [3]. The only treatment that offers a potential cure is complete resection of the tumor. However, in most of countries, the majority of patients are diagnosed at advanced stages and have a poor prognosis. Though first-line chemotherapy for advanced gastric cancer (AGC) prolongs overall survival (OS) and improves quality of life compared with best supportive care (BSC). The median survival of advanced gastric cancer patients who received palliative chemotherapy is approximately 7 to 11 months [4-8]. In 2010, trastuzumab (Herceptin, Roche, Basel, Switzerland), a recombinant humanized monoclonal antibody that targets human epidermal growth factor receptor-2 (HER2), had already been confirmed to be effective when combined with chemotherapy in HER2-positive AGC patients [9]. Trastuzumab is the first targeted agent that gets the indication in AGC, but it is not the only targeted agents which have tried their applications in AGC. The other therapeutic strategies include: epidermal growth factor receptor (EGFR) inhibitors, vascular endothelial growth factor (VEGF) inhibitors, hepatocyte growth factor (HGF) and its receptor c-MET pathway inhibitor, mammalian target of rapamycinm (m-TOR) inhibitor et al. This review will focus on the recent progress in targeted agents for the treatment of AGC (Table 1).

**Table 1:** Available targeted agents in advanced gastric cancer

**NCT code/trial name**	**Drugs**		**Setting**	**Masking**	**Primary endpoint**	**Arm**	**Status**	**Results**
PhaseIIIToGA/NCT01041404	HER2 inhibitor	Trastuzumab	First line	Double blind	OS	Cisplatin + fluoropyrimidine + Trastuzumab vs. Placebo	Finished	Positive
PhaseIIINCT01774786		Pertuzumab	First line	Double blind	OS	Trastuzumab + cisplatin + fluoropyrimidine + Pertuzumab vs. Placebo	Ongoing	Unknown
Phase III/IIINCT01641939		T-DM1	Second line	Open label	OS	T-DM1 vs. Taxane	Ongoing	Unknown
PhaseIII TYTAN/NCT00486954		Lapatinib	Second line	Open label	OS	Lapatinib vs. Paclitaxel	Finished	Negative
PhaseIII LOGiC/NCT00680901		Lapatinib	First line	Double blind	OS	Capecitabine + oxaliplatin + Lapatinib vs. Placebo	Finished	Negative
PhaseIIIEXPAND/NCT00678535	EGFR Inhibitors	Cetuximab	First line	Open label	PFS	Capecitabine and cisplatin + Cetuximab vs. Placebo	Finished	Negative
PhaseIIIREAL-3/NCT01234324		Panitumumab	First line	Open label	Frequency of pT3/T4 categories after surgery	EOX (epirubicin/oxaliplatin/capecitabine)	Ongoing	Unknown
PhaseIII AVAGAST/NCT00548548	VEGF inhibitors	Bevacizumab	First line	Double blind	OS	Capecitabine + cisplatin + Bevacizumab vs. Placebo	Finished	Negative
PhaseIII REGARD/NCT00917384		Ramucirumab	Second line	Double blind	OS	and BSC + Ramucirumab vs. Placebo	Finished	Positive
PhaseIIIRAINBOW/NCT01170663		Ramucirumab	Second line	Double blind	OS	Paclitaxel + Ramucirumab vs. Placebo	Ongoing	Unknown
PhaseIIINCT01246960		Ramucirumab	First line	Double blind	PFS	mFOLFOX6+ Ramucirumab vs. Placebo	Ongoing	Unknown
PhaseIIINCT00970138		Apatinib	Third line	Double blind	PFS	Placebo vs.Apatinib	Finishes	Positive
PhaseIIINCT01512745		Apatinib	Third line	Double blind	PFS	placebo vs. apatinib	Ongoing	Unknown
PhaseIIINCT01747551		Aflibercept	First line	Double blind	PFS	mFOLFOX6 + Aflibercept vs. Placebo	Ongoing	Unknown
PhaseIIINCT01152645	c-MET inhibitor	Tivantinib (ARQ 197)	Second/Thirdline	Open label	DCR	Tivantinib	Finished	Modest efficacy
PhaseIIINCT01662869		Onartuzumab	First line	Double blind	OS	mFOLFOX6 + Onartuzumab vs. Placebo	Ongoing	Unknown
PhaseIIIGRANITE-1/NCT00879333	m-TOR inhibitor	Everolimus (RAD001)	Second/third line	Double blind	OS	Everolimus vs. Placebo	Finished	Negative

*Abbreviation*: *HER2* human epidermal growth factor receptor-2, *OS* overall survival, *EGFR* epidermal growth factor receptor, *PFS* progression free survival, *VEGF* vascular endothelial growth factor, *BSC* best supportive care, *DCR* disease control rate, *m-TOR* mammalian target of rapamycinm.

### EGFR-signaling pathway

EGFR exists on the cell surface and is part of the family of TK receptor including HER2. EGFR overexpression has been reported in approximately 30% to 50% of gastric cancers and is associated with poor prognosis [10-14].

### Anti-HER2 monoclonal antibodys

HER2, a transmembrane tyrosine kinase (TK) receptor, is the preferred heterodimerization partner of the other HER family members (HER1 or EGFR, HER3 and HER4). The HER2-HER3 heterodimer plays a critical role in oncogenic transformation in HER2-driven tumors [15,16]. In breast cancer, amplification and overexpression of the HER2 gene is associated with poor outcomes, higher mortality, and higher recurrence as well as metastasis rate [17-19]. However, the association between HER2 status and prognosis in gastric cancer remains controversial. In a few studies a correlation between HER2 amplification or overexpression and favorable survival was shown [20,21]. In a retrospective analysis from four Chinese clinical centers, the HER2 status of 726 gastric cancer patients with all stages was detected. They found that HER2 was not a prognostic factor for gastric cancer patients [22]. A systematic analysis of data from the literature indicated that there was a clear trend towards a potential role for HER2 as a negative prognostic factor in gastric cancer patients [23].

### Trastuzumab

In the ToGA trial, the addition of trastuzumab to chemotherapy significantly improved OS compared with chemotherapy alone in patients with HER2-positive AGC. The HER2 positive rate was 22.1% in this study. The median OS was improved significantly in the trastuzumab arm compared with the control arm [13.5 vs. 11.1 months, P = 0.0048; hazard ratio (HR), 0.74; 95% confidence interval (CI), 0.60 to 0.91]. In subgroup analysis, the patients with HER2 immunohistochemistry (IHC) 2+/fluorescence in situ hybridization + or IHC 3+ had a longer OS compared with the chemotherapy-alone arm (16 months vs. 11.8 months). Moreover, the addition of trastuzumab to chemotherapy in the ToGA trial was well-tolerated, with no differences in the incidence of grade 3 or 4 adverse events (AEs) between the two groups [9]. Based on the result of ToGA study, trastuzumab with chemotherapy was recommended for HER2 overexpressing AGC (category 1 for combination with cisplatin and fluoropyrimidine; category 2B for combination with other chemotherapy agents; not recommended for use with anthracyclines) in the national comprehensive cancer network guidelines of gastric cancer version 2 2012.

### Pertuzumab

Pertuzumab is a monoclonal antibody that prevents dimerization of HER2 with other HER family receptors [24]. Its efficacy in combination with trastuzumab in HER2-positive metastatic breast cancer patients has been demonstrated in phase III clinical trials [25,26]. At the 2013 American Society of clinical oncology (ASCO) meeting, the result of an international phase III study was reported. This was a double-blind, placebo-controlled, randomized phase III study and it was designed to evaluate efficacy and safety of pertuzumab + trastuzumab + chemotherapy in patients with HER2-positive metastatic gastric or gastroesophageal junction (GEJ) cancer. In this study, HER2-positive metastatic gastric cancer patients were randomized 1:1 to receive trastuzumab + cisplatin + fluoropyrimidine + pertuzumab vs. placebo . Primary endpoint was OS. The result of this study is worth looking forward to [27].

### T-DM1

T-DM1 is an antibody-drug conjugate in which trastuzumab is conjugated to a cytotoxic compound, emtansine [28]. Upon binding of the trastuzumab moiety to HER2, T-DM1 is internalized into the tumor cell, releasing the DM1 moiety, which inhibits microtubules. A trial (NCT01641939) is now ongoing to examine the efficacy and safety of T-DM1 compared with standard taxane therapy in patients with HER2-positive gastric cancer in second line setting. In this study, patients will be randomized to one of the three groups, 3.6 mg/kg T-DM1 every 3 weeks, 2.4 mg/kg T-DM1 every week, or standard taxane therapy (Taxol 80 mg/m^2^/wk or docetaxol 75 mg/m^2^ q3wk), for at least four cycles (12 weeks). Planned endpoints include OS.

The results of these studies are eagerly awaited to examine the efficacy of this approach in patients with HER2-positive AGC.

### HER-2 TKI

Lapatinib, a reversible dual TKI that affects both HER2 and EGFR, has also been clinically shown to be active against HER2-positive breast cancer as a monotherapy and in combination with capecitabine [29]. The TYTAN study compared lapatinib and paclitaxel with paclitaxel alone in patients with HER2-positive metastatic gastric cancer in a second line setting. The primary endpoint of the study was OS. Though lapatinib showed efficacy in HER2 IHC 3+ subgroups, this study did not show an improvement in OS. These results indicated that the definition of HER2-positive AGC was very important for the development of new anti-HER2 agents [30]. At the 2013 ASCO meeting, lapatinib in combination with capecitabine plus oxaliplatin in HER2-positive advanced or metastatic gastroesophageal adenocarcinoma, the LOGiC trial was reported. Primary endpoint of improving OS was not met. The median OS in the lapatinib group and the control group was 12.2 months and 10.5 months, respectively, P = 0.3492. Improved OS was seen in Asian patients and patients under 60 years old. Secondary efficacy endpoints of progression free survival (PFS), response rate (RR) and duration of response were improved with addition of lapatinib. No new safety signal was identified, but increased toxicity was seen with the addition of lapatinib to capecitabine and oxaliplatin, particularly diarrhea and skin toxicity [31]. Prior to this study, there was no evidence to support the use of lapatinib in HER2-positive AGC.

### Cetuximab

Manageable and expected safety profiles with substantial activities were reported in phase II studies of cetuximab plus various first line chemotherapy regimens in patients with AGC [32-35].

In a prospective multi-center biomarker-oriented phase III trial using cetuximab with folfiri as first-line treatment in AGC, patients received weekly cetuximab (400 mg/m^2^ on day 1, subsequently 250 mg/m^2^) plus irinotecan and a 24-hour continuous infusion of folinic acid and 5-fu on days 1, 8, 15, 22, 29 and 36 of a 50-day cycle, until disease progress (PD). In 48 assessable patients, the overall response rate was 46% and disease control rate (DCR) was 79%. Median PFS and OS were 9.0 months (95% CI 7.1-15.6) and 16.5 months (95% CI 11.7-30.1), respectively. No obvious cetuximab-related side effects were recorded in this study. However, despite a favorable result in these phase II trials, the results of a randomized phase III trial comparing cetuximab in combination with capecitabine and cisplatin with chemotherapy alone (EXPAND) were negative. In this study, 904 patients were randomly assigned (1:1) to receive first-line chemotherapy (capecitabine and cisplatin) with or without cetuximab (400 mg/m^2^ on day 1, subsequently 250 mg/m^2^). The primary endpoint was PFS. The median PFS and OS were 4.4 and 9.4 months, respectively, in patients assigned to cetuximab plus chemotherapy compared with 5.6 and 10.7 months in patients receiving chemotherapy, respectively (PFS, p = 0.3158; OS, p = 0.9547) [36]. Addition of cetuximab provided no additional benefit to chemotherapy alone in the first-line treatment of advanced gastric cancer.

### Panitumumab

Panitumumab is a fully human, immunoglobulin G2 monoclonal antibody directed against the EGFR. In metastatic colorectal cancer, panitumumab has monotherapy activity in patients with chemotherapy refractory diseases and, when added to chemotherapy, can improve PFS in Kirsten-ras (KRAS) oncogene wide type patients in the first line and second line settings.

The REAL-3 trial was designed to compare combination chemotherapy (epirubicin/oxaliplatin/capecitabine, EOX regimen) with or without panitumumab in 553 patients with advanced esophagogastric adenocarcinoma at 63 centers. Median OS in 275 patients allocated to EOX was 11.3 months (95% CI 9.6-13.0) compared with 8.8 months (95% CI 7.7-9.8) in 278 patients allocated panitumumab group (HR 1.37, 95% CI 1.07-1.76; P = 0.013). Panitumumab group was associated with increased incidence of grade 3–4 diarrhoea, rash, mucositis and hypomagnesaemia but reduced incidence of haematological toxicity [37].

Based on the results above, no more clinical trials of anti-EGFR monoclonal antibody in gastric cancer will be carried out. Moreover, neither erlotinib nor gefitinib showed activity in anti-gastric cancer in phase III studies.

### VEGF-signaling pathway

Tumor angiogenesis and metastasis are strongly linked with angiogenesis in most solid tumors. VEGF is a key mediator of physiologic and pathologic angiogenesis [38].

### Anti-VEGF monoclonal antibody

Preclinical studies showed that bevacizumab (Avastin, Genentech/Roche, San Francisco, CA/Basel, Switzerland), a monoclonal antibody targeting VEGF-A, resulted in tumor growth inhibition when given as monotherapy or in combination with cytotoxic agents [39]. In patients with gastric cancer, VEGF expression has been linked to tumor aggressiveness and poor prognosis [14,40-42]. Several phase II trials combining bevacizumab with different chemotherapeutic compounds were conducted on treatment-naïve or pretreated patients with AGC or GEJ cancers [43-45]. AVAGAST was a prospective, random-assignment, double-blind, placebo-controlled phase III clinical trial comparing capecitabine/cisplatin with or without bevacizumab as first-line therapy in 774 patients with AGC [46]. The primary endpoint was OS. Although AVAGAST did not reach its primary endpoint (OS: 12.1 vs. 10.1 months for the bevacizumab and placebo groups (HR, 0.87; P = 0.1002)), both median PFS (6.7 v 5.3 months; HR, 0.80; 95% CI, 0.68 to 0.93; P = 0.0037) and RR (46.0% v 37.4%; P = 0.0315) were significantly improved with bevacizumab versus placebo. The incidence of grades 3 to 4 AEs potentially related to bevacizumab was similar in both arms.

### Anti-VEGFR monoclonal antibody

Ramucirumab (IMC-1121B; Im Clone Systems, New York, NY) is a fully human immunoglobulin G1 monoclonal antibody that binds with high affinity to the extracellular VEGF-binding domain of VEGFR-2. Phase I clinical trials demonstrated its objective antitumor activity and antiangiogenic effects over a wide range of dose levels, suggesting that ramucirumab may have a favorable therapeutic index in treating malignancies amenable to VEGFR-2 inhibition [47].

REGARD study [48] is a phase III, randomized, double-blinded trial of ramucirumab in the treatment of AGC or GEJ adenocarcinoma in second line setting. Patients were randomized 2:1 to receive ramucirumab (8 mg/kg IV) plus BSC or placebo plus BSC every 2 weeks until PD, unacceptable toxicity, or death. The primary endpoint was OS. The HR for OS was 0.776 (95% CI, 0.603-0.998; P = 0.0473). Median OS was 5.2 months for ramucirumab group and 3.8 months for placebo group. The HR for PFS was 0.483 (95% CI, 0.376-0.620; p < 0.0001). Median PFS was 2.1 and 1.3 months, 12-weeks PFS was 40% and 16%, ORR was 3.4% and 2.6% for ramucirumab and placebo groups respectively. The most frequent grade ≥ 3 AEs were: hypertension, anemia, abdominal pain, ascites and fatigue. Ramucirumab conferred a statistically significant benefit in OS and PFS compared to placebo in AGC in the second line setting with an acceptable safety profile. The phase III trial RAINBOW is a randomized multicenter double-blind, placebo controlled trial evaluating the safety and efficacy of paclitaxel plus ramucirumab drug product compared to paclitaxel plus placebo. This study is still on-going. Another randomized phase II clinical trial of mFOLFOX6 plus ramucirumab vs. placebo for AGC is also ongoing (Table 1).

### VEGFR TKI

Apatinib (YN968D1) is a small-molecular TKI agent targeting at VEGFR [49]. In a randomized, double-blind, multicenter, phase II, three-arm, placebo-control study of apatinib in patients with metastatic gastric carcinoma, patients were randomized to receive placebo or apatinib (placebo vs. apatinib at 850 mg once a day vs. 425 mg twice a day) [50]. The primary endpoint was PFS. The respective survival rates were as follows: median PFS, 1.4 months vs. 3.4 months vs. 3.4 months; median OS, 2.5 months vs. 4.8 months vs. 4.3 months. Common AEs included hypertension and hand-foot syndrome. Based on this result, a randomized, double blinded, placebo controlled multicenter phase III study in a third-line setting in AGC is currently being conducted in China. Patients are scheduled to receive apatinib 850 mg qd or placebo until PD or intolerable toxicity or patients’ withdrawal of consent. The primary endpoint is PFS (NCT01512745).

### VEGF trap

Aflibercept (VEGF Trap, Sanofi, Paris France; and Regeneron, Tarrytown, NY, USA) is a recombinant fusion protein consisting of extracellular domains of the human VEGFR fused to the Fc portion of human immunoglobulin G1 [51-53]. Its biological affinity for VEGF is reported to be significantly higher than that of bevacizumab [54]; however, there are no preclinical data to suggest its increased efficacy in AGC.

Aflibercept has recently been approved by the Food and Drug Administration for patients with treatment-resistant colorectal cancer. A phase II clinical trial to test the safety and effectiveness of aflibercept in combination with mFOLFOX6 compared to mFOLFOX6 alone in patients with AGC is ongoing.

### Other targeted agents

#### MET inhibitor

c-Met is a proto-oncogene that encodes a protein known as hepatocyte growth factor receptor (HGFR) [55,56]. It stimulates cell scattering, invasion, protection from apoptosis and angiogenesis [57]. Upon HGF stimulation, MET induces MET kinase catalytic activity which triggers transphosphorylation of the tyrosine (Tyr) 1234 and Tyr 1235, thus initiating a whole spectrum of biological activities driven by MET. A high level of c-Met expression has been correlated with poor survival in patients with gastric cancer [58].

c-Met inhibitors include monoclonal antibodies and small molecules that inhibit the enzymatic activity of the c-Met TK. There are basically two classes of c-Met inhibitors, ATP competitive and ATP non-competitive inhibitor. ATP competitive inhibitors are further divided into two classes; class I (SU-11274-like) and class II (AM7-like) on the basis of different types of binding and a third group of noncompetitive ATP inhibitor that binds in a different way to the other two [59,60].

Elevated expressions of c-MET and its ligand, HGF, have been frequently found in gastric cancer, and are associated with a more aggressive disease [61,62]. Tivantinib is a selective, non-ATP competitive, small-molecule inhibitor of c-MET and is under development in several cancers. In a single-arm phase II study, the efficacy of tivantinib monotherapy in Asian patients with previously treated AGC was evaluated [63]. Tivantinib was daily administered 360 mg bid orally. The primary endpoint was DCR. No objective response was observed, and DCR was 36.7% (11/30 patients). Median PFS was 43 (95% CI: 29.0-92.0) days. There is no treatment-related death and novel safety concern. Tivantinib as a monotherapy showed a modest efficacy in previously treated AGC, and further trial testing in combination would be warranted in AGC.

Onartuzumab is a humanized mAb directed against HGFR. A randomized, phase III, multicenter, double-blind, placebo-controlled study evaluating the efficacy and safety of onartuzumab in combination with mFOLFOX6 in patients with metastatic Her2-negative, c-Met-positive gastroesophageal cancer is now ongoing.

### m-TOR inhibitor

mTOR is a key protein kinase that regulates cell growth and proliferation, cellular metabolism and angiogenesis [64]. Mutations in these components result in inappropriate mTOR activation [64]. The mTOR pathway has been shown to be frequently dysregulated in a variety of human cancers, including gastric cancer [65]. Overexpression of the mTOR downstream effectors eIF4E and 4E binding protein 1 (4E-BP1) was shown in GI cancer cells [64]. Everolimus (RAD001) is an oral inhibitor of m-TOR, which is downstream of the Akt pathway. Everolimus reduced 4E-BP1 phosphorylation and attenuated production of the proangiogenic factors hypoxia-inducible factor and VEGF in these gastric cancer cell lines [65]. Everolimus has demonstrated antitumor activity in gastric cancer in preclinical studies [64-66] and a phase I study involving patients with AGC [67]. The results of a phase IIstudy of everolimus in 53 patients with previously treated AGC showed a disease control rate of 56.0% and median PFS of 2.7 months. At a median follow-up duration of 9.6 months, the median OS was 10.1 months and good tolerability was observed [68]. Based on this promising result, a prospective phase III trial of everolimus in previously treated patients with advanced gastric cancer: GRANITE-1 was conducted [69]. A total of 656 patients from 23 countries were enrolled; 439 were randomized to everolimus, 217 to placebo. Median OS was 5.39 months with everolimus vs 4.34 months with placebo (HR, 0.90; 95% CI, 0.75-1.08; P = 0.1244). Median PFS was 1.68 vs 1.41 months (HR, 0.66; 95% CI, 0.56-0.78; p < 0.0001). The most common grade 3/4 adverse events were anemia, decreased appetite and fatigue. Everolimus monotherapy did not significantly improve OS in patients with AGC previously treated with 1 or 2 lines of systemic chemotherapy.

### Future perspectives

Emerging data from the development of targeted therapy have provided novel strategies that are expected to translate into survival benefits for AGC patients. The results of the ToGA study recently demonstrated that the addition of trastuzumab to chemotherapy can bring survival benefit to HER2-positive AGC patients. However, this benefit is limited to only 20% of AGC patients (HER2-positive). Therefore, there remains a critical need for both the development of more effective agents and the identification of predictive molecular markers to select those patients who might benefit most from specific targeted therapies. Till now both cetuximab and bevacizumab have failed in phase III trail for AGC. The results of pertuzumab and T-DM1 are worth waiting for. The other promising targeted agents include: ramucirumab, aflibercept and apatinib as well as c-MET inhibitors.

## Abbreviations

AGC: Advanced gastric cancer; OS: Overall survival; BSC: Best supportive care; HER2: Human epidermal growth factor receptor-2; EGFR: Epidermal growth factor receptor; VEGF: Vascular endothelial growth factor; HGF: Hepatocyte growth factor; mTOR: Mammalian target of rapamycinm; TK: Tyrosine kinase; HR: Hazard ratio; CI: Confidence interval; IHC: Immunohistochemistry; AEs: Adverse events; GEJ: Gastroesophageal junction; OS: Overall survival; PFS: Progression free survival; ASCO: American Society of clinical oncology; PFS: Progression free survival; RR: Response rate; PD: Disease progress; DCR: Disease control rate; KRAS: Kirsten-ras; HGFR: Hepatocyte growth factor receptor; Tyr: Tyrosine.

## Competing interests

The authors declare that they have no competing interests.

## Authors’ contributions

All authors have contributed to data preparation, drafting and revising the manuscripts. Both authors have read and approved the final manuscript.
